# Prognostic role of long noncoding RNA CASC11 in cancer patients: A meta-analysis

**DOI:** 10.1097/MD.0000000000040823

**Published:** 2024-12-13

**Authors:** Song Zhang, Bo Xu, Ji-Ling Zhang, Shun-Hai Liu, Xin Xiang, Pan Liu

**Affiliations:** a Department of Hepatic-Biliary-Pancreatic Surgery, The Fourth People’s Hospital of Neijiang, Sichuan, China; b Department of Hepatic-Biliary-Pancreatic Surgery, The First People’s Hospital o Neijiang, Sichuan, China.

**Keywords:** cancer, CASC11, long noncoding RNA, meta-analysis, prognosis

## Abstract

**Background:**

Long noncoding RNA (lncRNA) is a significant component of the noncoding genome and refers to RNA molecules that exceed 200 nucleotides in length. It plays a crucial role in both promoting and suppressing cancer by regulating the proliferation, invasion, and metastasis of tumor cells. We have found a correlation between cancer susceptibility candidate 11 (CASC11) experssion and tumorigenesis development prognosis in a number of studies on tumors. Hence, this meta-analysis was conducted to further investigate the effects of CASC11 expression on clinicopathological features and outcome. Furthermore, CASC11 can act both as a therapeutic target and a cancer biomarker.

**Method:**

We conducted a thorough search of PubMed, Embase, Web of Science, and Cochrane Library to identify all eligible studies until September 20, 2023. These studies examined the potential relationship between the expression levels of CASC11 and survival or the range of pathological feature in cancer patients. The impact of CASC11 expression on overall survival (OS) was evaluated using pooled hazard ratios and 95% confidence intervals (CI). The relationship between CASC11 expression and clinicopathological features was assessed through pooled odds ratios and 95% CI.

**Results:**

A total of 11 studies, involving 660 patients, were examined in this analysis. The results revealed that the overexpression of CASC11 was significantly associated with poor OS (hazard ratios = 2.07, 95%CI = 1.64–2.60) in cancer. Further subgroup analysis demonstrated that the overexpression of CASC11 was consistently linked to poorer OS in diverse types of cancer, including digestive system neoplasm, respiratory neoplasms, and gynecologic tumor.

**Conclusion:**

Overall, this analysis established a strong correlation between CASC11 expression and tumor prognosis, suggesting its potential as a predictive marker for tumor progression in diverse cancer types.

## 1. Introduction

In recent times, the rate of cancer-related fatalities continues to rise, along with the incidence of cancer itself. The number of new cancer patients has reached 18.1 million, and cancer-related death was reported to be 9.6 million in 2018.^[1]^ The main cancers that lead to death are lung cancer, female breast cancer, prostate cancer, colorectal cancer, and stomach cancer.^[2]^ Various approaches such as surgery, chemotherapy, and targeted therapy have been employed in cancer treatment. However, the outcomes have not satisfactory so far.

Long noncoding RNA (lncRNA), shares similarities with microRNA but does not have the ability to code for proteins,^[3]^ and the length of lncRNA is usually over 200 nucleotides.^[4]^ In recent years, there has been a growing focus on studying the role and mechanisms of lncRNA in cancer. Abnormal expression of lncRNA has been observed in several types of cancer, where it influences tumor development by interacting with downstream signaling molecules involved in regulating gene expression. For instance, Zhang et al discovered that upregulation of lncRNA MALAT1 was associated with the growth and prognosis clear cell carcinoma of kidney.^[5]^ Moreover, another report suggested that reduction in expression of lncRNA XIST and JPX was associated with the significance of lncRNA in tumor progression has garnered attention, particularly in terms of its potential for cancer diagnosis when its expression is dysregulated.

Cancer susceptibility candidate 11 (CASC11) is a type of lncRNA, which is located on humans chromosomes 8q24.21.^[6]^ It has been observed to be overexpressed in various cancers such as cervical cancer, stomach cancer, esophagus cancer, liver cancer, lung cancer, glioma, and prostate cancer. This suggests that CASC11 may have a role in promoting tumor formation. In some studies, CASC11 has been found to regulate tumor growth, invasion, metastasis, and apoptosis through its interaction with different microRNAs, carcinogenic proteins, carcinogens, and transcription factors.^[7]^ In addition, CASC11 has also been implicated in coronary heart disease and osteoporosis.^[8–20]^ Recent studies have revealed abnormal expression of CASC11 in different solid tumors and its involvement in cell proliferation, metastasis, invasion, apoptosis, and chemotherapy resistance, all of which have significant implications for tumor prognosis. Therefore, CASC11 could potentially serve as a biomarker and therapeutic target for cancer. So, identification of a reliable biomarker for tumor diagnosis is crucial, as it holds significant potential for enhancing the efficacy of tumor treatment. Hence, this study aimed to evaluate and predict the overall risk of survival prognosis and clinicopathological features in patients with malignant tumors through a meta-analysis of relevant articles.

## 2. Materials and methods

### 2.1. Literature search

An electronic search was performed in PubMed, EMBASE, Web of science, and Cochrane Library for all the relevant studies which reported the association between lncRNA CASC11 expression and clinical outcomes in different cancers by utilizing the following search strings: “long noncoding RNA CASC11” or “lncRNA CASC11” or “CASC11.” These terms were used in different combinations. The most recent search was conducted on September 20, 2023. And the language restrictions is English. Additionally, relevant literature was manually examined to gather further research findings.

### 2.2. Inclusion and exclusion criteria

Inclusion criteria: (1) studies needed to be cohort studies; (2) subjects had to have a confirmed diagnosis of malignant tumor based on histopathology, and the language restrictions is English; (3) the studies needed to demonstrate the prognostic role of CASC11 in patients with various types of cancer; (4) studies that divided patients into high and low expression groups based on the level of CASC11 expression; (5) studies that provided sufficient information to estimate the hazard ratio (HR) or odds ratio (OR), and its 95% confidence intervals (CI).

Exclusion criteria: (1) studies with overlapping or duplicated data; (2) letters, case reports, reviews, and preclinical studies; (3) studies lacking adequate information on survival rates.

### 2.3. Data extraction and quality assessment

Two researchers independently gathered pertinent data using specific criteria for inclusion and exclusion. Any disagreements were resolved through discussion with a third investigator. The extracted information from each study included the last name of the first author, publication year, cancer type, patient count, outcome measures, CASC11 expression cutoff value, method of obtaining HR, pathology details (e.g., gender, tumor differentiation, lymph node metastasis, tumor size, and TNM stage), as well as the HRs and 95% CIs representing the expression of CASC11 for overall survival (OS) and disease-free survival (DFS).

In order to ensure the quality of the study, 2 researchers independently evaluated the literature’s quality by utilizing the Newcastle-Ottawa Scale (NOS). The NOS comprises specific scoring criteria such as the representativeness of the exposed and nonexposed groups, determination of the exposure group, absence of outcome events in participants prior to study commencement; determination of exposure group; evaluation of outcome events, adequacy of follow-up time, and integrity of follow-up. The fifth factor accounted for 2 points, while the others were weighted as 1 point, resulting in a total score of 9 points. A NOS score of 6 or higher was deemed to be indicative of high quality.

### 2.4. Statistical methods

ORs with 95%CI were estimated to evaluate the correlation between CASC11 expression and the clinical outcomes in cancer patients. Statistical analysis was performed by utilizing Revman 5.0 software and Stata 14. Cochrane *Q* test and *P* values were used to determine the heterogeneity across the studies. If heterogeneity was present (I^2^ ≥ 50% or *P* < .05), random-effect model was used to pool the results. Conversely, the fixed-effect model was applied for the analysis. As for the acquisition of hazard rations of survival, the data were directly extracted from original articles. If not applicable, the data were calculated by using Engauge Digitizer version 4.1 from Kaplan–Meier curves. The publication bias was evaluated utilizing funnel plot, and *P* < .05 was considered as the existence of publication bias.

All analyses were based on previous published studies, thus no ethical approval and patient consent are required.

## 3. Result

### 3.1. Basic information of the included studies

**Figure 1.**
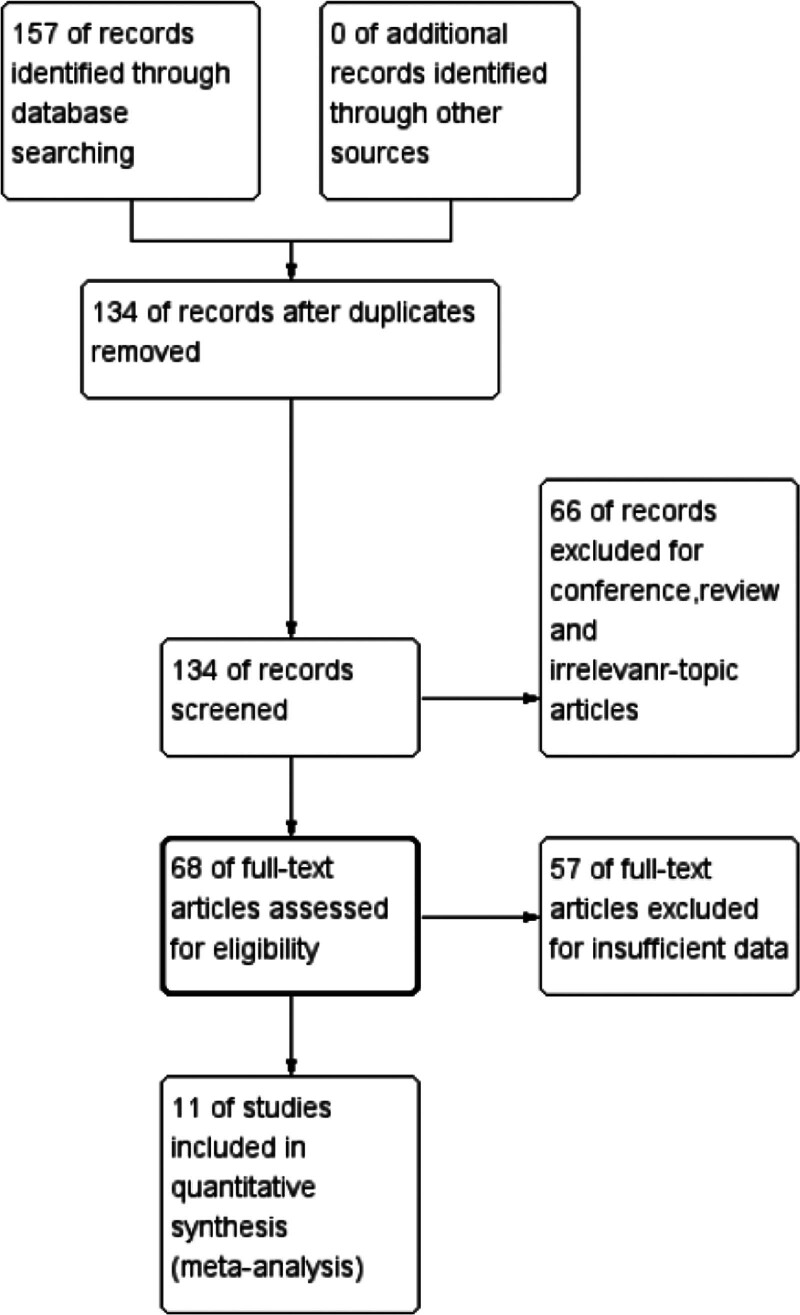
Flowchart presenting the steps of literature search and selection.

A total of 157 reports were obtained through preliminary literature search. However, 146 were excluded based on the literature search process as depicted in Figure 1, and 11 studies were finally included. Finally, a total of 11 reports were included and thus sufficient data available for conducting a meta-analysis.^[10–20]^ A total of 660 patients were diagnosed with various malignant tumors based on pathology comprising of liver, esophagus, lung, ovarian, colorectal, and cervical cancer. The publication time of the included literatures was 2016 to 2023. The sample size of each study ranged from 36 to 78, with the qRT-PCR method being used for detection.

A total of 10 studies evaluated the potential relationship between the high and low expression of CASC11 and OS,^[10–19]^ and 1 study explored the association between CASC11 and DFS,^[18]^ and 7 studies provided information about the different clinicopathological parameters.^[10,11,15,17–20]^ NOS scores of all the studies were ≥ 6, and the specific basic characteristics of the included literatures have been shown in Table 1.

**Table 1:** Characteristics of CASC11 studies in the meta-analysis.

Author	Country	Year	Type	Sample	Number	HER	Cutoff value	Outcome	Survival analysis method	HR (95%CI)	Follw-wp time (month)	NOS
Chen F. et al	China	2021	HCC	Tissue	72	50%	Median	OS, CF	Single factor analysis	2.76 (1.17–6.49)	80	8
S.G. Chen et al	China	2019	EC	Tissue	45	37.50%	Not mentioned	OS, CF	Not mentioned	2.01 (0.83–4.85)	80	9
N. Cheng et al	China	2019	HCC	Tissue	68	48.50%	Jorden index	OS	Single factor analysis	2.03 (0.89–4.63)	60	9
Y. Cui et al	China	2020	OC	Tissue	64	53.10%	Jorden index	OS	Single factor analysis	1.87 (0.93–3.78)	60	9
Y. Fu et al	China	2019	SCLC	Plasma	71	52.10%	Jorden index	OS	Single factor analysis	1.90 (0.95–3.79)	60	9
X. Gu et al	China	2023	LC	Plasma	60	50%	Median	OS, CF	Not mentioned	2.29 (1.20–4.37)	36	9
Y. Han et al	China	2019	HCC	Tissue	76	47.40%	Median	OS	Single factor analysis	1.97 (0.99–3.89)	80	9
W. Hsu et al	China	2019	CC	Tissue	50	50%	Median	OS, CF	Single factor analysis	1.82 (0.84–3.94)	40	9
H. Song et al	China	2020	HCC	Tissue	78	50%	Median	OS, DFS, CF	Single factor analysis	1.93 (1.01–3.67)	60	8
R. Yan et al	China	2019	NSCLC	Tissue	40	57.50%	Not mentioned	OS, CF	Single factor analysis	1.77 (0.73–4.29)	60	8
Z. Zhang et al	China	2016	CRC	Tissue	36	50%	Median	CF	Not mentioned	Not mentioned	Not mentioned	8

CC = cervical cancer, CF = clincopathologic feature, CI = confidence intervals, CRC = colorectal cancer, DFS = disease-free survival, EC = esophagus cancer, HCC = hepatocellular carcinoma, HER = high expression ratio, HR = hazard ratios, LC = lung adenocarcinoma cell, NOS = Newcastle-Ottawa Scale, NSCLC = non-small cell lung cancer, O = ovarian cancer, SCLC = small cell lung cancer.

### 3.2. Meta-analysis results

#### 3.2.1. lncRNA-CASC11 expression and OS

**Figure 2.**
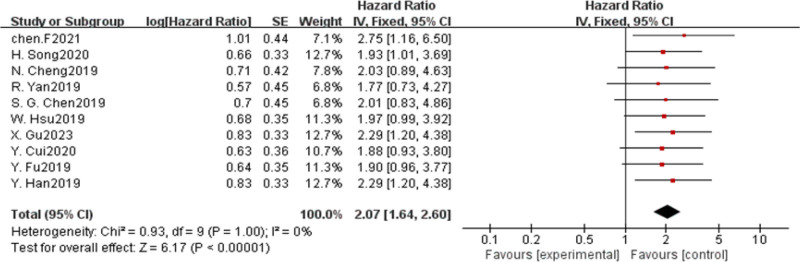
Forest plots of the association between CASC11 expression of patients with cancer. CASC11 = cancer susceptibility candidate 11.

Ten studies were conducted to analyze the possible relationship between CASC11 expression level and OS.^[10–19]^ There was no heterogeneity observed among the various studies (I^2^ = 0%, *P* = 1). Therefore, fixed-effect model was employed for quantitative analysis. It was found that elevated CASC11 expression was significantly associated with poor OS (HR = 2.07, 95%CI = 1.64–2.60) in various malignancies (Fig. 2). As only one of the included report mentioned DFS, only descriptive analysis was carried out. Interestingly, it was noted that cancer patients with high CASC11 expression had worse DFS. Further subgroup analysis was performed to assess various factors such as cancer type, method used to obtain hazard ratio, sample size, and the cutoff value for CASC11 expression.

**Figure 3.**
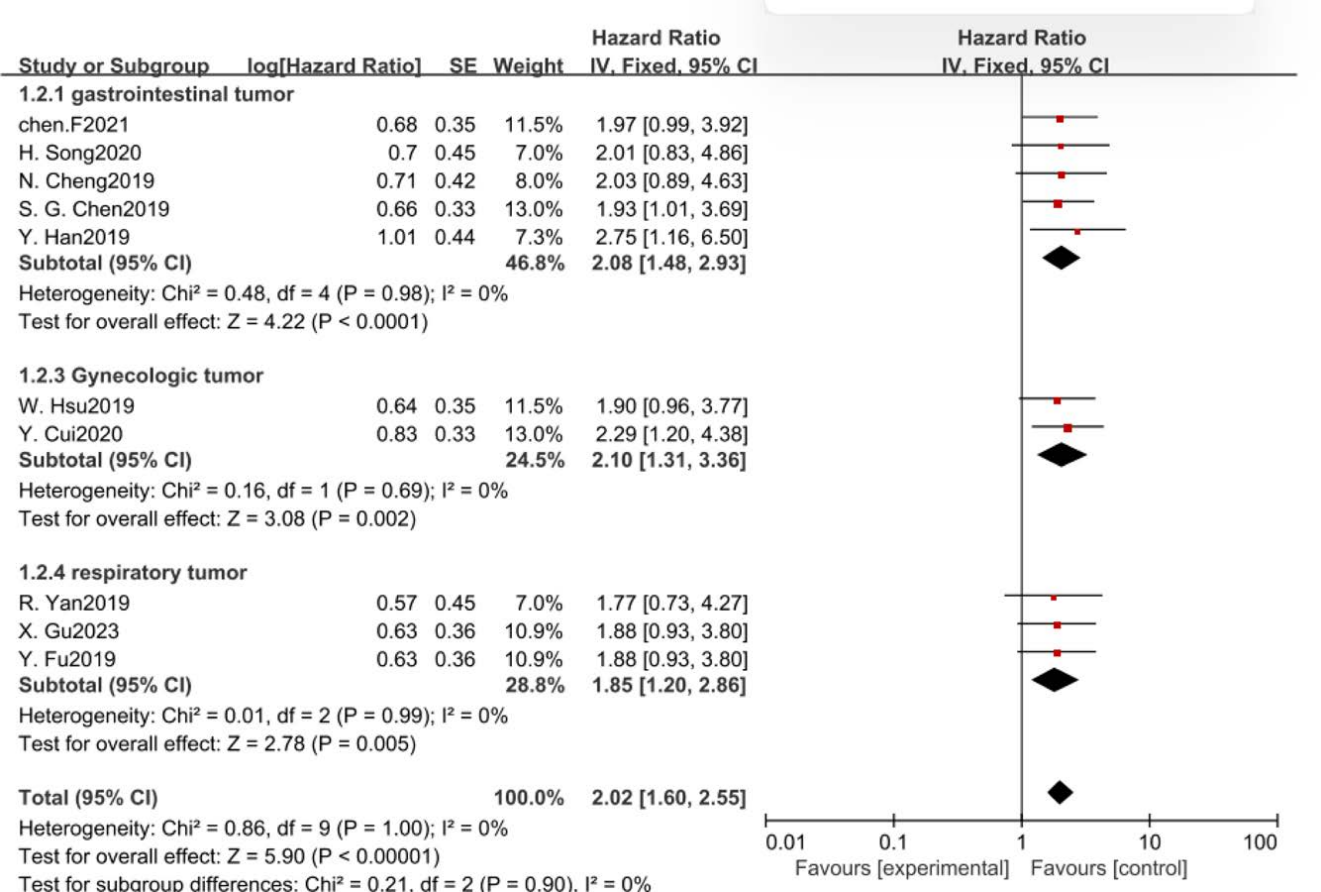
Forest plot of HRS for the association between CASC11 expression and OS in subgroup analysis based on different cancer types. CASC11 = cancer susceptibility candidate 11, OS = overall survival.

As shown in Table 1, a total of 8 different tumor types were collected, including liver, esophageal, colorectal, small cell lung, non-small cell lung, lung adenocarcinoma, cervical, and ovarian cancers. So we divided these tumors into 3 subgroups, digestive system tumors, respiratory system tumors, and gynecological tumors. Since there was no heterogeneity among the subgroups, a fixed model was used for analysis, our data suggested that increased CASC11 expression in gastrointestinal tumor, respiratory neoplasms, and gynecologic tumor was statistically associated with shorter OS (Table 2, Fig. 3).

**Table 2:** The results of subgroup analysis.

Subgroup	Number of studies	Number of patients	HR (95%CI)	*P*	Model	Heterogeneity	
						*P*	I^2^
*Method*							
Single	8	480	1.97 (1.52–2.56)	.00001	Fixed	1	0
Else	2	144	2.19 (1.30–3.69)	.00001	Fixed	.82	0
*Sample size*							
≥60	6	135	2.09 (1.56–2.81)	.00001	Fixed	.99	0
<60	3	489	1.86 (1.15–3.02)	.00001	Fixed	.98	0
*Tumor type*							
GT	5	339	2.08 (1.48–2.93)	.00001	Fixed	.98	0
RN	3	171	1.85 (1.20–2.86)	.00001	Fixed	.99	0
Gynecologic tumor	2	114	2.10 (1.31–3.36)	.00001	Fixed	.69	0
*Cutoff value*							
Medain	5	336	2.10 (1.53–2.88)	.00001	Fixed	.96	0
Else	5	288	1.91 (1.35–2.71)	.00001	Fixed	1	0

GT = gastrointestinal tumor, HR = hazard ratios, RN = respiratory neoplasms.

**Figure 4.**
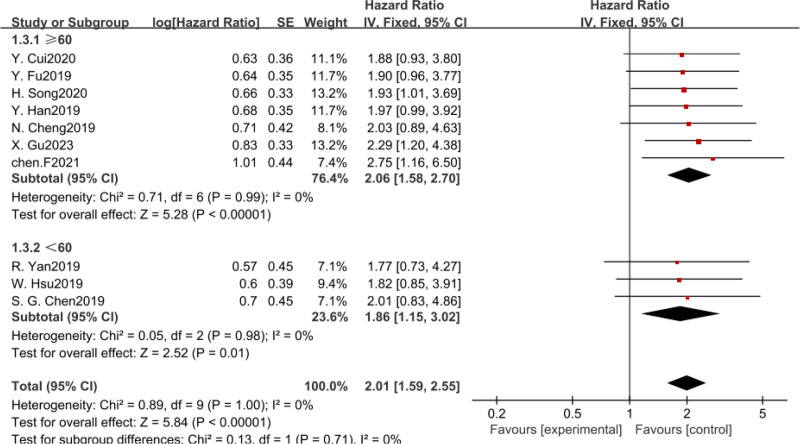
Forest plot of HRS for the association between CASC11 expression and OS in subgroup analysis based on sample sizes. CASC11 = cancer susceptibility candidate 11, OS = overall survival.

**Figure 5.**
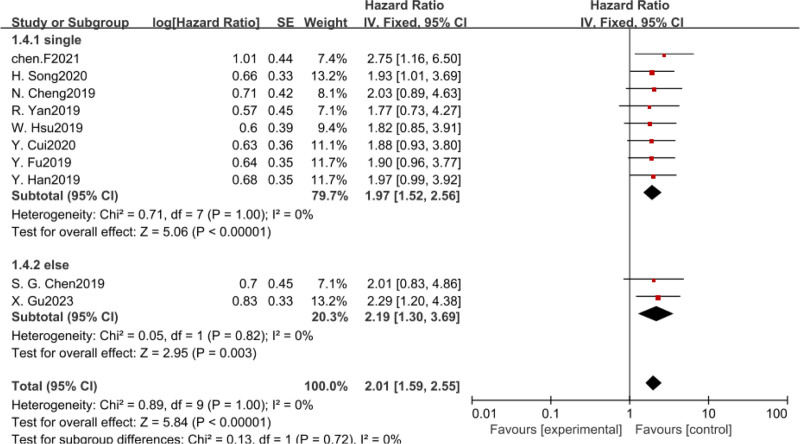
Forest plot of HRS for the association between CASC11 expression and OS in subgroup analysis based on survival analysis method. CASC11 = cancer susceptibility candidate 11, OS = overall survival.

**Figure 6.**
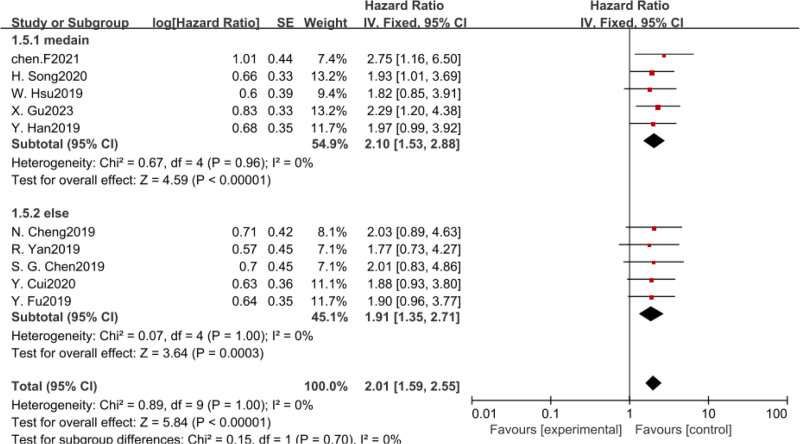
Forest plot of HRS for the association between CASC11 expression and OS in subgroup analysis based on cutoff value. CASC11 = cancer susceptibility candidate 11, OS = overall survival.

In addition, we also conducted subgroup analysis according to intercept value, sample size, and survival analysis method. There was no heterogeneity among the subgroups, and fixed-effect model was used for analysis. The results revealed that CASC11 expression was significant with poor OS in all subgroup meta-analysis (Table 2, Figs. 4–6).

#### 3.2.2. Association of CASC11 expression and clinicopathological parameters

Seven studies provided effective clinicopathological characteristics and conducted a detailed quantitative analysis.^[10,11,15,17–20]^ The statistical results indicated that there was no association between the expression level of CASC11 and age, sex, tumor size, or lymph node metastasis. However, higher levels of CASC11 expression were more likely to predict worse staging (OR = 3.52, 95%CI = 1.89–6.56) and distant metastasis (OR = 4.07, 95%CI = 2.25–7.35) (Table 3). The high expression of CASC11 was significantly associated with distant metastasis and clinical stage (*P* < .05).

**Table 3:** The relationship between the expression level of CASC11 and clinicopathologic features in neoplasms.

Feature	Number of studies	Number of patients	OR (95%CI)	*P*	Model	Heterogeneity	
						*P*	I^2^
Age (old/middle)	5	259	1.61 (0.97–2.66)	.06	Fixed	.87	0
Sex (male/female)	6	331	1.01 (0.64–1.58)	.97	Fixed	.89	0
Tumor size (big/small)	5	286	1.34 (0.56–3.20)	.50	Random	.01	68%
TNM (I, II/III, IV)	4	181	3.52 (1.89–6.56)	.0001	Random	.59	0
Lymph node metastasis (yes/no)	5	259	0.54 (0.23–1.31)	.74	Random	.03	62%
Distant metastasis (yes/no)	4	219	4.07 (2.25–7.35)	.01	Fixed	.09	53%

CI = confidence intervals, OR = odds ratios.

### 3.3. Publication bias

**Figure 7.**
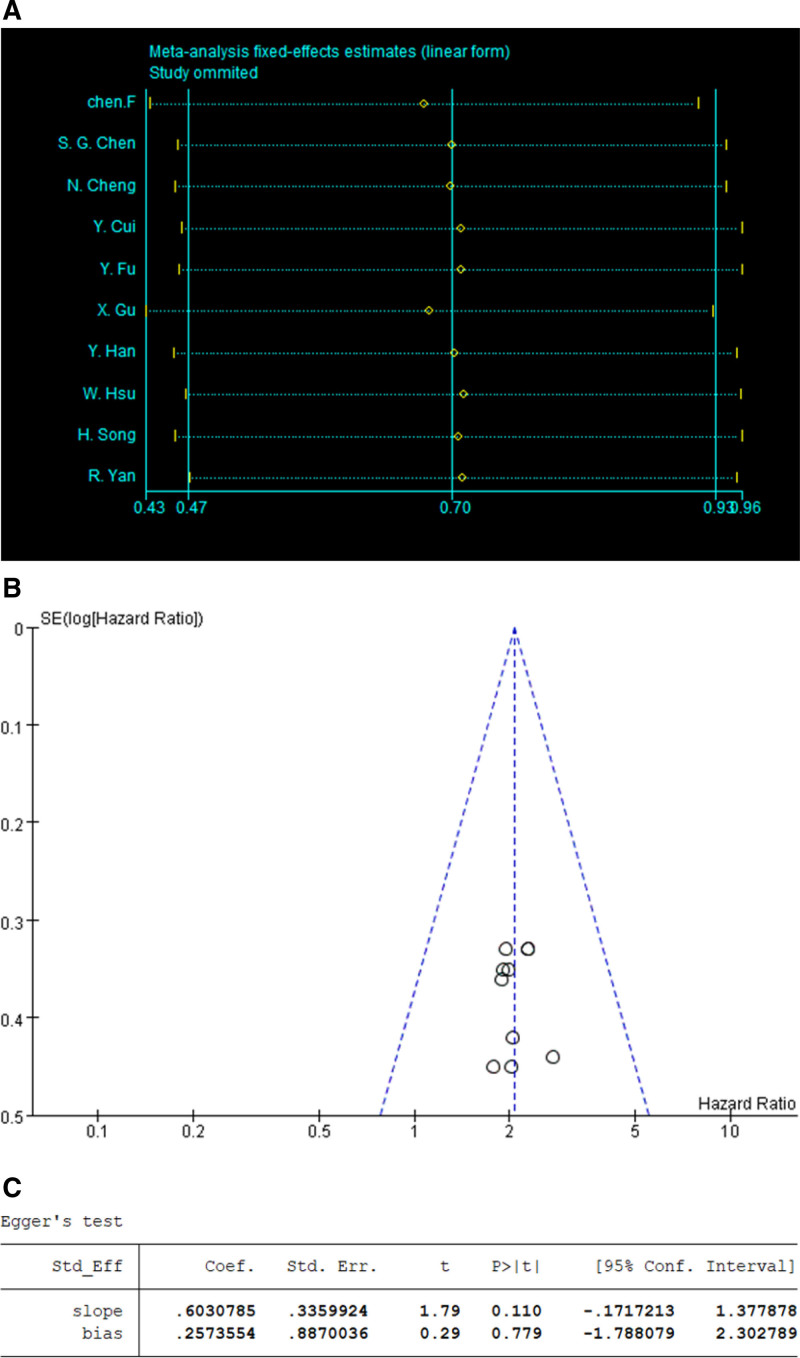
(A) Sensitivity analysis of OS. (B) Funnel plot of OS. (C) Egger test of OS. OS = overall survival.

Sensitivity analysis of the various outcome indicators was conducted by Stata software. The results indicated that the overall statistical effect did not change significantly even when individual studies were removed from each paper, indicating that the findings of this study were consistent as well as reliable (Fig. 7). Thereafter, in order to evaluate for publication bias in these studies, Begg funnel plot test was performed (Fig. 7). This result revealed that there was no significant publication bias in the meta-analysis for OS (*P* > .05) (Fig. 7). However, publication bias was not analyzed in the DFS as there were only a small number of studies available for evaluation.

## 4. Discussion

LncRNAs have emerged as a critical player in cancer, with a growing body of research confirming their involvement in various tumor-related biological processes. Numerous studies have demonstrated the significance of lncRNAs as an important factor under both physiological and pathological conditions.^[21]^ CASC11, a newly discovered type of lncRNA, has been observed to have abnormal expression in diverse types of cancer. It is also associated with poor prognosis, as its abnormal expression can promote tumor proliferation and metastasis. However, no previous studies have investigated the relationship between CASC11 and prognosis in cancer patients. This meta-analysis was performed to analyze the potential relationship between CASC11 expression level and prognosis of cancer patients by including relevant articles.

Recently, a large number of studies have demonstrated that CASC11 plays a crucial role in multiple signal transduction pathways, leading to a deeper understanding of its involvement in the occurrence and development of malignant tumors. For instance, Tong et al found that CASC11 was highly expressed in non-small cell lung cancer and could significantly promote the proliferation of A549 cells,^[22]^ primarily by targeting miR-302/CDK1. In another study, Cheng et al found that CASC11 exhibited a sponge-like function in facilitating the proliferation of liver cancer cells through miR-188-5P.^[12]^ In addition, Zhang et al found that that CASC11 can play a direct role in activating the Wnt/β-catenin signaling pathway through its interaction with ribonucleoprotein K.^[20]^ This pathway has significant implications in the progression and growth of colorectal cancer. Furthermore, the study revealed a positive association between high levels of CASC11 expression and key indicators of tumor development, such as the tumor size, serous infiltration, lymph node metastasis, and TNM stage. Moreover, similar mechanisms exist in other tumors, which are not discussed here. Furthermore, other studies have shown that high CASC11 expression can markedly improve the drug resistance of malignant tumors, whereas knockdown CASC11 can reduce the drug resistance of cancer cells to chemotherapy drugs and improve the sensitivity of cancer cells to chemotherapy drugs.^[23]^ Overall, all these studies indicate that CASC11 could act as a poor prognostic indicator of malignant tumors, which was consistent with the results of this study.

Tumor diameter, size, lymph node metastasis, and distant metastasis are regarded as key indicators to determine tumor stage and prognosis of patients.^[24]^ Subsequently, this study further evaluated the potential relationship between CASC11 and various clinical characteristics of patients with malignant tumors. The results revealed that the high expression of CASC11 was significantly associated with distant metastasis and staging. It has been suggested that the high expression of CASC11 is a key factor in the poor prognosis of cancer patients. However, due to different tumor diameters and age boundaries in the included studies, this report used middle-aged/elderly and large/small tumors as the grouping criteria, without a clear definition of values, which could affect the accuracy of the results.

This meta-analysis also has a few limitations. First, all the patients included in the study were Chinese, so the predictive value of CASC11 for malignant tumor patients in other countries and regions requires further investigation. Second, the number of reports included in this paper is relatively small, which could potentially impact the accuracy of the results. Third, some of the original data from literature was not directly available, but obtained through the calculation of survival curve. There could be some errors in the process of data extraction, which can adversely affect the accuracy of the results. Fourth, CASC11 expression level in the literature reviewed was not given a precise value, but was divided into high and low levels. Lastly, the study encompassed a broad range of tumor types across various systems, and there were limited literature resources focusing on specific tumors or systems. All of these factors may have influenced the final results of the study.

In conclusion, the findings of this meta-analysis revealed that the elevated expression of lncRNA CASC11 could serve as a negative factor when assessing the clinical outcome of cancer patients. Furthermore, upregulation of lncRNA CASC11 could act as a predictor of distant metastasis and advanced stage of the disease. These results suggested that CASC11 could be used as a valuable predictive biomarker for both prognosis and clinical pathology. However, further research with a larger sample size is required to validate our findings, allowing CASC11 to be widely applied clinically.

## Author contributions

**Conceptualization:** Song Zhang, Bo Xu, Pan Liu.

**Data curation:** Shun-Hai Liu, Ji-Ling Zhang.

**Formal analysis:** Shun-Hai Liu.

**Investigation:** Xin Xiang.

**Project administration:** Xin Xiang.

**Writing – original draft:** Song Zhang, Bo Xu.

**Writing – review & editing:** Pan Liu.
